# Syndecan-2 enhances invasive motility in colon cancer cells *via* a laminin-332–integrin α6β1 axis

**DOI:** 10.1016/j.jbc.2026.113455

**Published:** 2026-08-17

**Authors:** Subin Cho, Jisun Hwang, Eunhye Park, Hyeonju Yang, Masumi Matsunuma, Keisuke Hamada, Yuji Yamada, Yamato Kikkawa, Eok-Soo Oh

**Affiliations:** 1 Department of Life Sciences, Ewha Womans University, Seoul, South Korea; 2 Laboratory of Cellular Biochemistry, School of Pharmacy, Tokyo University of Pharmacy and Life Science, Hachioji, Tokyo, Japan

**Keywords:** adhesion, cancer, extracellular matrix, integrin, laminin-332, syndecan-2

## Abstract

Syndecan-2 (SDC-2) is a key regulator of colon cancer progression, but its cooperation with integrins in cell motility is not fully understood. Here, we show that SDC-2 overexpression in HT29 colon cancer cells (HT29-S2W) enhances adhesion, spreading, and migration on laminin-332, an extracellular matrix component linked to poor prognosis. These effects correlate with increased basal membrane localization of integrin α6, as well as elevated expression and deposition of laminin-332 subunits (α3, β3, and γ2), thereby promoting laminin-332 assembly within the extracellular matrix. Proximity ligation assays revealed enhanced interactions between SDC-2 and integrin α6β1 following SDC-2 overexpression, indicating coordinated signaling mediated by laminin-332. Notably, HT29-S2W cells adhered more efficiently to full-length laminin-332 containing the SDC-2-binding LG4/5 modules of the laminin α3 chain than to truncated laminin-332 lacking these modules. Furthermore, both an anti-integrin α6 antibody and a laminin-α3-derived peptide (A3G75aR) significantly inhibited adhesion to full-length laminin-332. In metastatic DLD-1 cells, which express high levels of SDC-2 and laminin-332, combined treatment with the antibody and peptide markedly suppressed cell migration and invasion. Collectively, these findings identify an SDC-2–laminin-332–integrin α6β1 axis that drives invasive motility and may represent a promising therapeutic target in metastatic colorectal cancer.

Syndecans (SDCs) are a family of type I transmembrane heparan sulfate proteoglycans, comprising four members (SDC-1 to -4), that function as cell surface receptors by interacting with a wide array of extracellular ligands through their glycosaminoglycan chains (1). These interactions allow SDCs to participate in diverse signaling pathways that govern key cellular processes such as proliferation, adhesion, migration, and differentiation (2). A primary mechanism by which SDCs exert their function is through binding to components of the extracellular matrix (ECM) and soluble growth factors, thereby influencing cytoskeletal dynamics and adhesion-mediated signaling (3, 4).

Among the syndecan family, syndecan-2 (SDC-2) is of particular interest due to its overexpression in various cancers, especially colon cancer, where it functions as both an adhesion receptor and docking molecule, contributing to tumor progression and metastasis (5, 6, 7, 8). Despite its recognized importance, the precise molecular mechanisms by which SDC-2 modulates ECM interactions and actin cytoskeleton organization during cancer progression remain incompletely understood. Notably, overexpression of SDC-2 enhances the spreading of HCT116 human colon carcinoma cells on fibronectin (9). In addition, the LG4/5 domain in the α3 subunit of laminin-332 promotes cell adhesion and migration through interactions with both SDC-1 (10) and SDC-2 (11). Furthermore, enhanced expression of laminin γ2 and β3 chains at the invasive front of carcinoma cells has been associated with poor prognosis in colon cancer (12), highlighting the potential role of SDC-2 in laminin-332-mediated tumor invasion.

Integrins represent another major class of cell adhesion receptors that play critical roles in tissue development, immune response, and cancer progression (13). Several integrins, including α6β4 and α1β1, have been implicated in colon cancer progression through pathways involving focal adhesion kinase, Src, and JNK signaling (14, 15).

Emerging evidence highlights a functional interplay between SDCs and integrins in regulating cytoskeletal architecture and tumor cell behavior (16). For instance, SDC-1 promotes cell migration and survival by cooperating with IGF1-R and activating integrins αvβ3 and αvβ5 (17), while SDC-4 regulates PKCα and focal adhesion formation in conjunction with integrin α5β1 (18). SDC-2 has also been shown to enhance integrin-dependent stress fiber formation and migration in several cancer cell models, including colon cancer, through cooperation with integrins such as α5β1 and α2β1 (19, 20). Furthermore, the extracellular domain of SDC -2 promotes β1 integrin-mediated adhesion *via* interaction with CD148 in endothelial cells (21).

Collectively, these findings suggest that SDC-2 may functionally coordinate with integrins to modulate ECM engagement and activate signaling pathways that drive tumorigenic processes. However, the molecular details of this interaction—particularly in the context of colon cancer cell motility and ECM interactions—remain to be fully elucidated. In this study, we investigated the potential cooperative role of integrins as co-receptors for SDC-2 in regulating ECM adhesion, cytoskeletal dynamics, and motility in human colon cancer cells.

## Results

### Laminin-332 serves as a major substrate for HT29 cell adhesion, primarily through integrins α3β1 and α6β1

**Figure 1:**
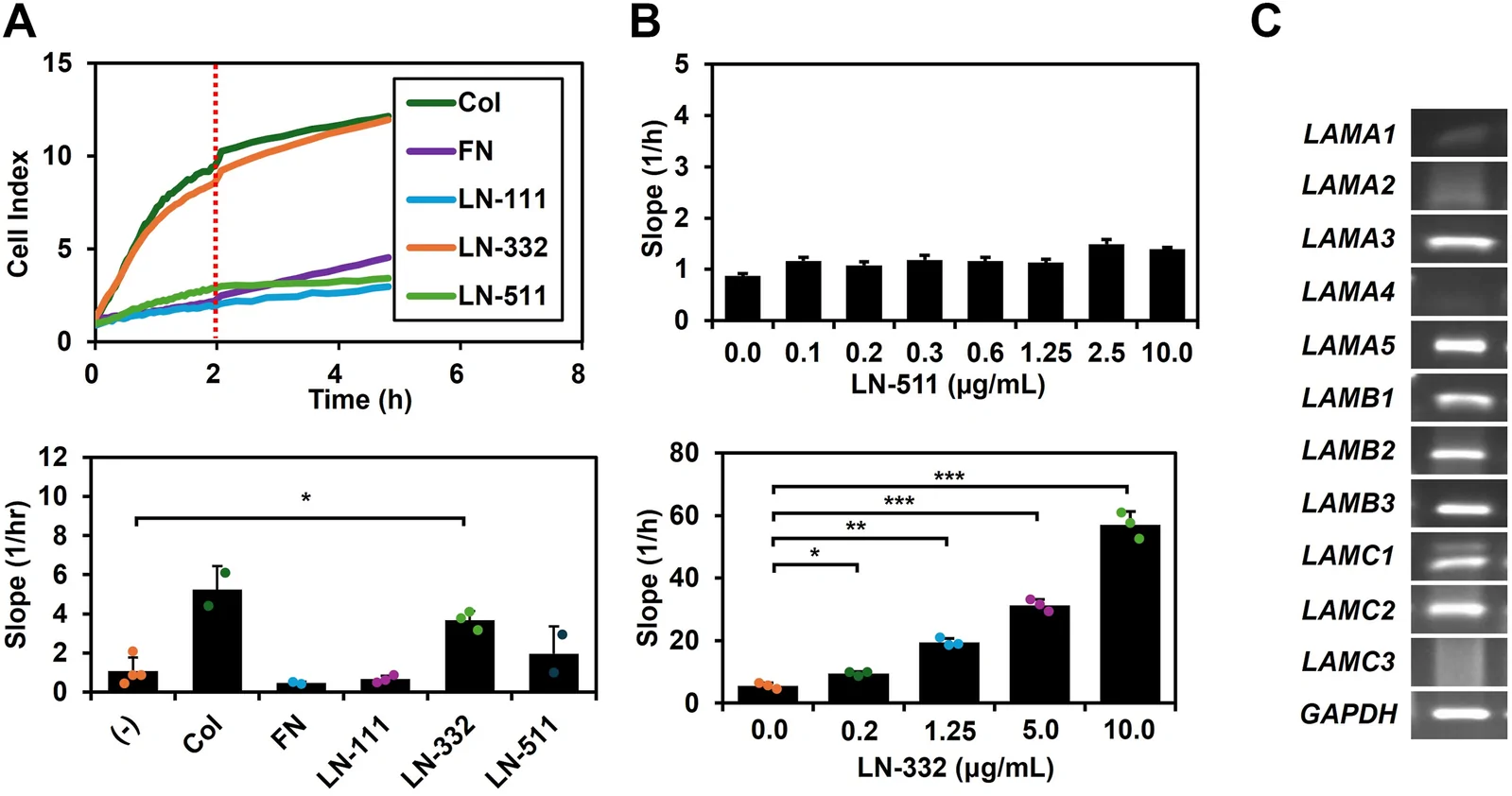
**Laminin-332 is a major substrate for HT29 colon cancer cell adhesion.***A*, HT29 cells (1.5 × 10^4^ cells/well) were seeded to E-plates coated with the indicated ECM with gelatin as a control. Cell adhesion was monitored using the xCELLigence system (*top panel*) and the rates of cell adhesion over 2 h were quantified using the RTCA software (*bottom panel*). ∗, *p* < 0.05 *versus* the no-ECM (−) condition. N = 2 to 4. *B*, HT29 cells (1.5 × 10^4^ cells/well) were seeded to E-plates coated with laminin-511 (LN-511) and laminin-332 (LN-332). Cell adhesion was monitored for 2 h using the xCELLigence system, and the rate of cell adhesion was quantified using RTCA software. Data are presented as mean ± S.D. from three independent experiments. ∗, *p* < 0.05; ∗∗, *p* < 0.01; ∗∗∗, *p* < 0.001 *versus* the no-ECM (0.0) condition. N = 2 (*top panel*) and N = 3 (*bottom panel*). *C*, total RNA was extracted from exponentially growing HT29 cells, and the mRNA expression levels of each laminin chain were analyzed by RT-PCR, using GAPDH as a loading control. ECM, extracellular matrix.

To investigate the regulatory role of SDC-2 in the tumorigenic activity of colon cancer cells, we first examined which ECM components support cell adhesion in HT29 human colon cancer cells. When plated on various ECM-coated surfaces, HT29 cells adhered most strongly to type I collagen, consistent with previous reports (20), followed by recombinant laminin-332 lacking the α3 LG4/5 domain, which mimics mature laminin-332 (22) (Fig. 1*A*). Notably, HT29 cells exhibited stronger and concentration-dependent adhesion to laminin-332 compared to laminin-511 and laminin-111 (Fig. 1*B*). RT-PCR analysis confirmed endogenous expression of all three laminin-332 subunits (α3, β3, and γ2) in HT29 cells (Fig. 1*C*), supporting the role of laminin-332—alongside type I collagen—as a key ECM component mediating HT29 cell adhesion.

**Figure 2:**
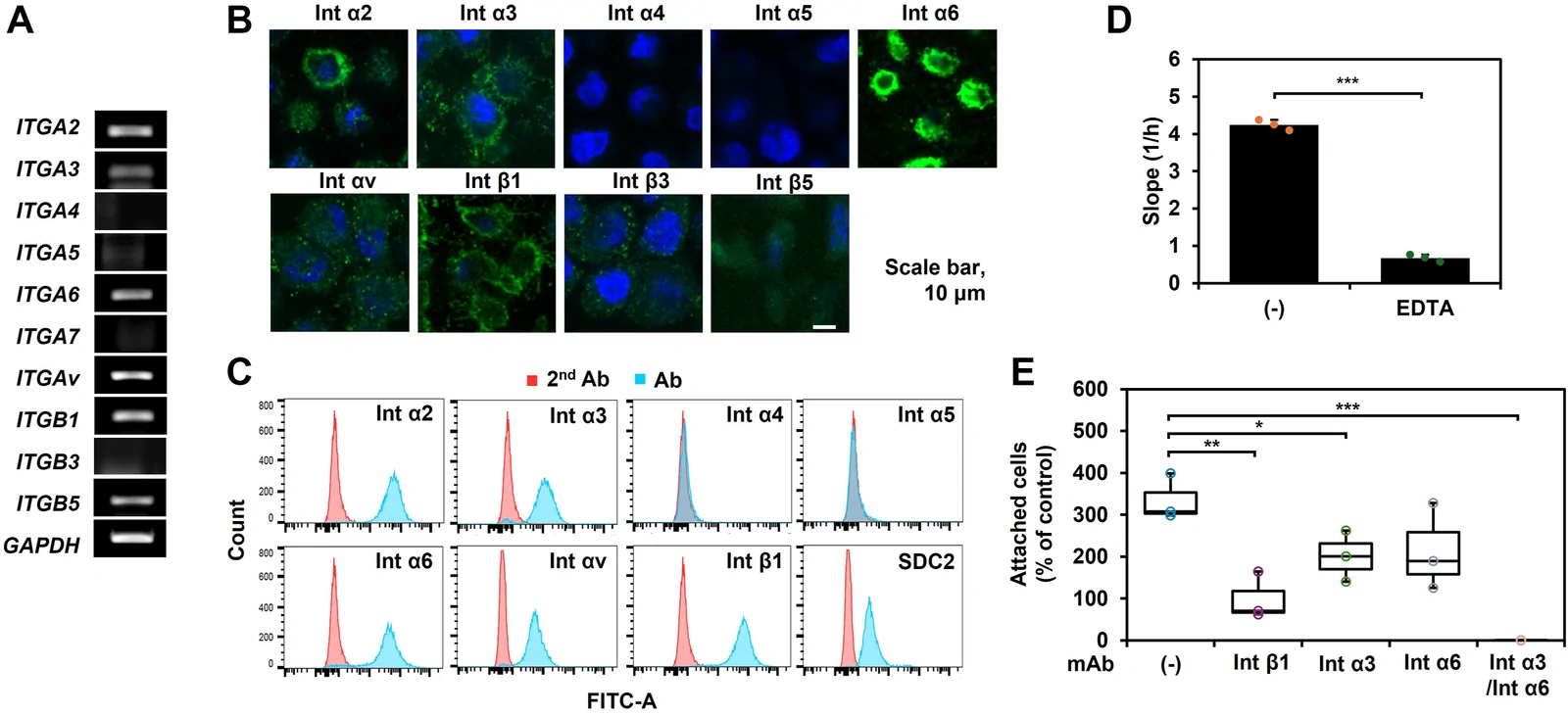
**Integrins α3β1 and α6β1 are primarily involved in the adhesion of HT29 cells to laminin-332.***A*, total RNA was extracted from exponentially growing HT29 cells, and the mRNA expression levels of each integrin subunit were analyzed by RT-PCR using GAPDH as a loading control. *B*, HT29 cells cultured on coverslips were immunostained with antibodies against specific integrin subunits and visualized with a FITC-conjugated goat anti-mouse antibody. DAPI was used to stain the nuclei. *C*, HT29 cells were analyzed by flow cytometry using either a monoclonal anti-SDC-2 antibody or antibodies against specific integrin subunits. IgG was used as a control. *D*, HT29 cells were seeded onto laminin-332-coated E-plates in the absence (−) or presence of 5 mM EDTA. Cell adhesion was monitored using the xCELLigence system. Data are presented as mean ± S.D. ∗∗∗, *p* < 0.001 *versus* the (−) condition. N = 3. *E*, HT29 cells were seeded into laminin-332-coated 96-well plates in the presence of the indicated monoclonal blocking antibodies against integrin subunits. After 1 h of incubation at 37 °C, the adhesive cells were stained and counted. Box-and-whisker plots show median, 25th and 75th percentiles, and minimum and maximum values (n = 3 independent experiments). Data are presented as mean ± S.D. ∗, *p* < 0.05; ∗∗, *p* < 0.01; ∗∗∗, *p* < 0.001 *versus* the no-antibody (−) condition. DAPI, 4′,6-diamidino-2-phenylindole; SDC-2, syndecan-2; FITC, fluorescein isothiocyanate.

Given that integrins are central mediators of cell adhesion to laminin family proteins (23), we analyzed the expression profiles of integrin subunits known to interact with laminin. RT-PCR analysis revealed robust expression of integrin subunits α2, α3, α6, αV, β1, and β5, as well as minimal expression of α5 and β3, while α4 and α7 were not detected (Fig. 2*A*). Immunofluorescence staining confirmed significant expression of α2, α3, α6, and β1, with particularly high levels of the α6 subunit (Fig. 2*B*). Flow cytometry further validated surface expression of integrins α2, α3, α6, αV, and β1 (Fig. 2*C*).

Considering that integrin α2β1 primarily binds type I collagen (24, 25) and integrins α6β1 and α3β1 are major receptors for laminin-332 (26), it is likely that HT29 cell adhesion to laminin-332 is mediated predominantly by α6β1 and α3β1. To directly assess the role of these integrins, HT29 cells were plated on laminin-332-coated surfaces in the presence of integrin inhibitors. Treatment with EDTA, a divalent cation chelator that inhibits integrin activation (27), significantly reduced cell adhesion by ∼75% to laminin-332 lacking the α3 LG4/5 domain (Fig. 2*D*). Blocking antibodies against specific integrin subunits showed that individual blockade of α6 or α3 reduced adhesion by approximately 40%, with the inhibition of adhesion being most pronounced by blockade of the β1 integrin and combined blockade of both α6 and α3 completely abolished HT29 cell adhesion to laminin-332 lacking the α3 LG4/5 domain (Fig. 2*E*). These findings confirm that integrins α6β1 and α3β1 are critical for HT29 cell adhesion to laminin-332.

### SDC-2 regulates HT29 cell interaction with laminin-332 *via* integrin α6β1

**Figure 3:**
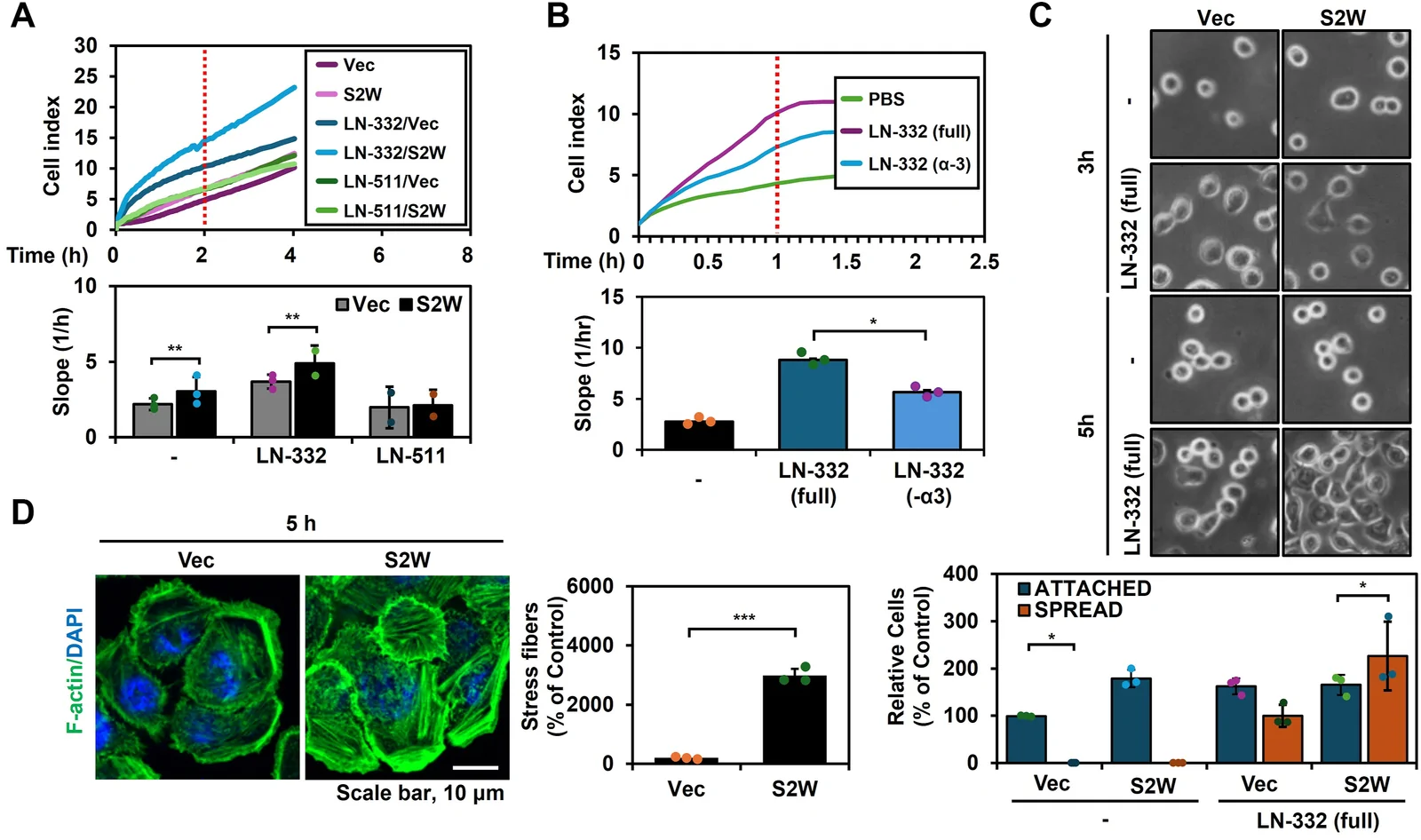
**Syndecan-2 enhances adhesion of HT29 cells to laminin-332.***A*, HT29-Vec and HT29-S2W cells (1.5 × 10^4^ cells/well) were seeded onto E-plates coated with full-length laminin-332 or laminin-511. Cell adhesion was monitored in real time using the xCELLigence system (*top panel*), and adhesion rates over 1 h (*B*) or 2 h (*A*) were quantified using RTCA software (*bottom panel*). ∗∗*p* < 0.01 *versus* control. N = 2 to 3. *B*, adhesion of HT29-S2W cells was assessed on full-length laminin-332 or a truncated form lacking the α3 LG4/5 domain, as described in (*A*). ∗*p* < 0.05 *versus* LN-332. N = 3. *C* and *D*, after cells (1.0 × 10^5^ cells/well in 24-well plates) were seeded into full-length laminin-332-coated plates, digital photographs were taken using a phase-contrast microscope at the indicated time points (*top panel*), and the adherent and spread cells were counted. Results are expressed as means ± SD of cell counts from three randomly selected fields (*bottom panel*, *C*). For stress fiber analysis (*D*), cells were fixed 5 h after plating and stained with FITC-conjugated phalloidin. Stress fiber formation was quantified by measuring the mean phalloidin fluorescence intensity per cell using Fiji (ImageJ) and is presented as mean ± SD ∗*p* < 0.05; ∗∗*p* < 0.01; ∗∗∗, *p* < 0.001 *versus* control. N = 3. FITC, fluorescein isothiocyanate.

SDC-2 is known to bind directly to the LG4/5 modules of laminin α3 chains (22), suggesting that SDC-2 may influence integrin–laminin interactions. To assess the contribution of SDC-2 to HT29 cell adhesion, we compared cells stably overexpressing SDC-2 (HT29-S2W) with vector-only controls (Vec) using full-length laminin-332, which contains the α3 LG4/5 module. When plated on full-length laminin-coated surfaces, both cell types adhered better to laminin-332 than to laminin-511, and HT29-S2W cells showed significantly increased adhesion to full-length laminin-332 (Fig. 3*A*). Consistently, HT29-S2W cells adhered more efficiently to full-length laminin-332 than to a truncated form lacking the α3 LG4/5 domain (Fig. 3*B*). Furthermore, after 5 h of incubation, HT29-S2W cells showed enhanced cell spreading on full-length laminin-332 (Fig. 3*C*) with increased actin stress fiber formation (Fig. 3*D*). Together, these findings indicate that SDC-2 enhances both adhesion and spreading of HT29 cells on full-length laminin-332.

**Figure 4:**
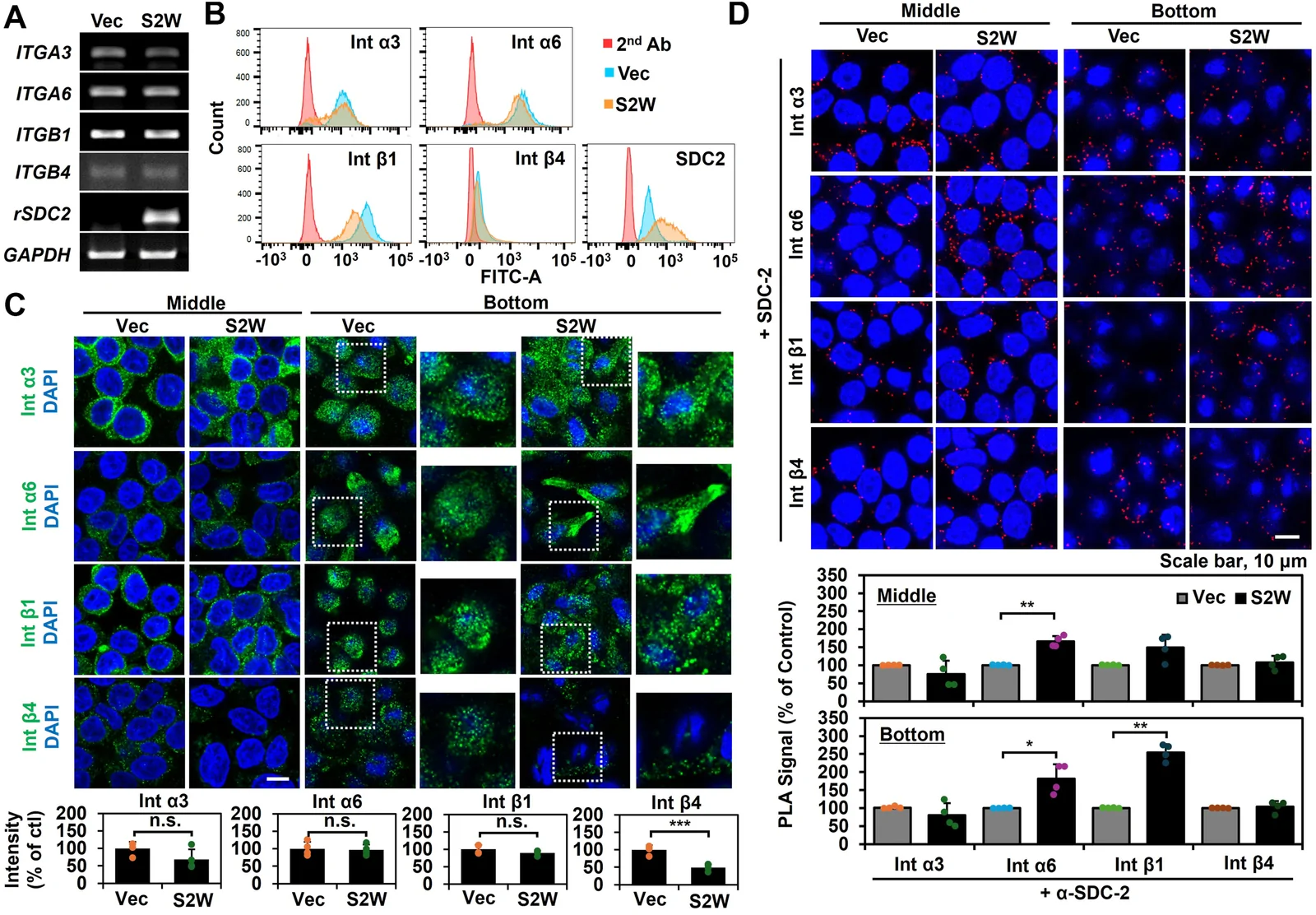
**Syndecan-2 interacts with integrin α6β1 at the basal cell layers.***A*, total RNA was extracted from exponentially growing HT29-Vec or HT29-S2W cells, and the mRNA expression levels of integrin (ITG) subunits and rat SDC-2 (rSDC2) were analyzed by RT-PCR, using GAPDH as a loading control. *B*, cells were analyzed by flow cytometry using specific antibodies against integrin subunits (Int) and a monoclonal anti-SDC-2 antibody. IgG was used as a control. *C*, ccultured on coverslips were immunostained with antibodies against integrin α3, α6, β1, or β4, followed by incubation with an FITC-conjugated goat anti-mouse secondary antibody. Nuclei were counterstained with DAPI. Fluorescence intensity was quantified from four randomly selected fields and is presented as the mean ± SD. ∗∗*p* < 0.001 *versus* HT29-Vec cells. N = 3. *D*, PLA assays were performed with the indicated antibodies. The data are presented as the relative number of PLA signals (*dots*) per cell and are expressed as the mean ± S.D. from four independent fields. ∗*p* < 0.05; ∗∗*p* < 0.01 *versus* HT29-Vec cells. N = 3. DAPI, 4′,6-diamidino-2-phenylindole; FITC, fluorescein isothiocyanate; PLA, proximity ligation assay.

Since integrins α6β1 and α3β1 are established receptors for laminin-332, we hypothesized that SDC-2 cooperates with these integrins to promote cell adhesion. To test this possibility, we examined whether SDC-2 overexpression affected the expression or subcellular localization of integrin subunits (Fig. 4). RT-PCR and flow cytometric analyses revealed no significant differences in the mRNA expression or cell surface levels of integrin α3, α6, or β1 between control and HT29-S2W cells (Fig. 4, *A* and *B*). However, immunofluorescence analysis showed that integrin α6 was preferentially redistributed to basal cell–ECM adhesion sites in HT29-S2W cells, whereas integrin α3 remained predominantly localized at lateral cell-cell junctions, similar to control cells (Fig. 4*C*). In contrast, integrin β4, which pairs with integrin α6 to form α6β4, was expressed at substantially lower levels than integrin β1 at both the mRNA and cell surface levels (Fig. 4, *A* and *B*). Moreover, SDC-2 overexpression further reduced basal localization of integrin β4 (Fig. 4*C*). Collectively, these findings suggest that SDC-2 enhances HT29 cell adhesion to laminin-332 by promoting the recruitment of integrin α6β1 to cell–ECM adhesion sites.

To further investigate the potential co-receptor relationship between SDC-2 and laminin-binding integrins, we performed proximity ligation assays (PLA) to assess the spatial proximity between SDC-2 and individual integrin subunits (Fig. 4*D*). SDC-2 overexpression significantly increased PLA signals with integrin α6 and β1, but not with integrin α3 or β4, compared with control cells. When the analysis was restricted to the basal cell surface, PLA signals for integrin α6 and β1 were further increased by 1.8-fold and 2.5-fold, respectively, whereas no significant changes were observed for integrin α3 or β4 (Fig. 4*D*). These findings indicate that SDC-2 preferentially associates with integrin α6β1 at basal cell–ECM adhesion sites, supporting a co-receptor mechanism through which SDC-2 promotes HT29 cell adhesion to laminin-332.

### SDC-2 enhances the expression and deposition of laminin-332 in the ECM of HT29 cells

**Figure 5:**
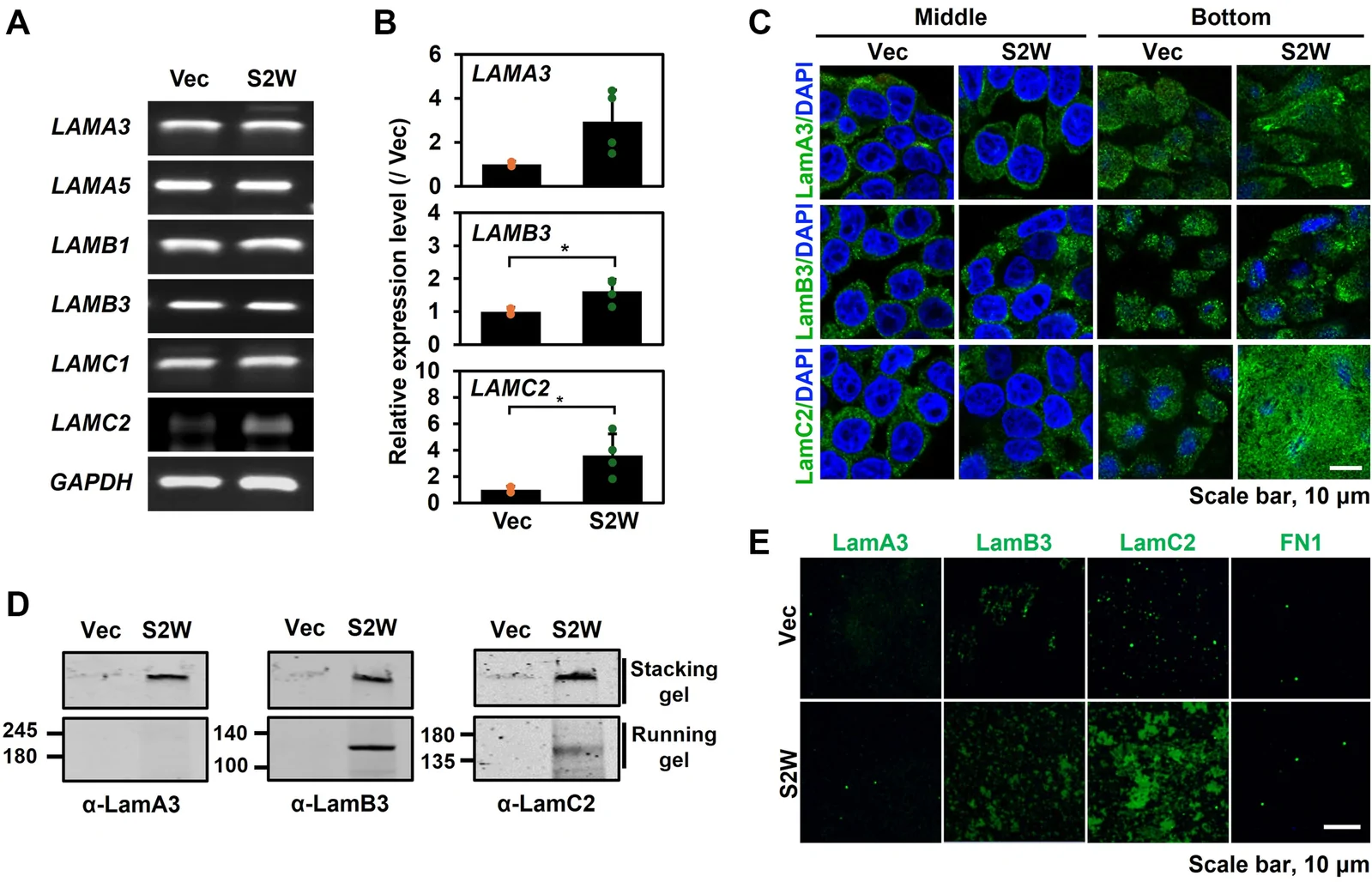
**Syndecan-2 enhances the expression and deposition of laminin-332.***A* and *B*, total RNA was extracted from exponentially growing HT29 or HT29-S2W cells, and the mRNA expression levels of laminin chains were analyzed by RT-PCR, using GAPDH as a loading control. *B*, laminin chain mRNA levels were further quantified by real-time PCR (qPCR) in three independent experiments. Data are shown as mean ± S.D. ∗*p* < 0.05; ∗∗*p* < 0.01 *versus* HT29-Vec cells. *C*, cells cultured on coverslips were immunostained with antibodies against the laminin α3 (LamA3), β3 (LamB3), or γ2 (LamC2) chains, followed by incubation with an FITC-conjugated goat anti-mouse secondary antibody. Nuclei were counterstained with DAPI. *D*, ECM proteins were isolated from HT29 cells by ammonium hydroxide extraction and analyzed by immunoblotting using antibodies against the indicated laminin chains. *E*, HT29 cells cultured on coverslips were decellularized using ammonium hydroxide. The cell-free ECM was immunostained with antibodies against the indicated laminin chains and subsequently visualized with an FITC-conjugated goat anti-mouse antibody. DAPI was used to stain nuclei. DAPI, 4′,6-diamidino-2-phenylindole; FITC, fluorescein isothiocyanate.

The organization of the ECM is tightly regulated by transmembrane receptors such as SDCs (28) and integrins (29). Increased laminin-332 expression is also associated with epithelial-mesenchymal transition and poor prognosis in various cancers, including colon cancer (30, 31, 32). Given these associations, we investigated whether SDC-2 affects the expression and deposition of laminin-332 in HT29 cells (Fig. 5). RT-PCR analysis showed that SDC-2 specifically upregulated the expression of the laminin γ2 chain (Fig. 5*A*). Consistent with these findings, qPCR analysis revealed that the γ2 chain exhibited the most pronounced increase among the laminin-332 subunits (Fig. 5*B*). Immunofluorescence analysis further confirmed that SDC-2 overexpression significantly increased laminin-332 protein levels, with a marked enrichment of the γ2 chain at the basal cell surface (Fig. 5*C*). To evaluate the deposition of laminin-332 in the ECM, we performed immunoblotting and immunofluorescence analyses after decellularization. We found that SDC-2 overexpression promoted the deposition of laminin α3, β3, and γ2 chains in the ECM (Fig. 5, *D* and *E*). This suggests that SDC-2 not only increased expression but also facilitated the incorporation of laminin-332 into the ECM.

**Figure 6:**
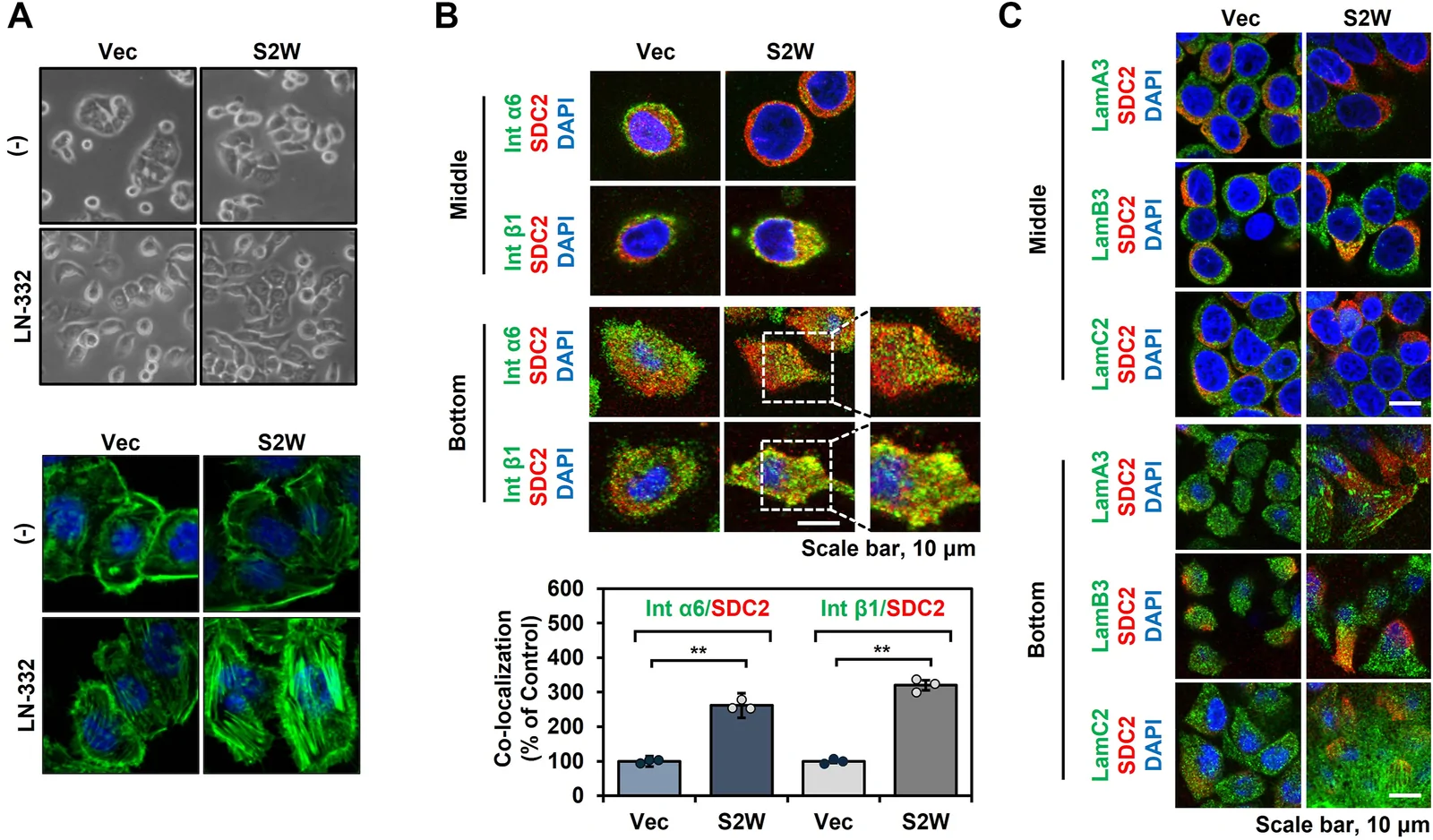
**Syndecan-2 promotes integrin α6β1 recruitment and adhesion complex formation on laminin-332.** HT29-Vec or HT29-S2W cells (1.0 × 10^5^ cells/well in 24-well plates) were seeded to full-length laminin-332-coated plates and incubated at 37 °C for 24 h. *A*, cells were digitally photographed under a phase contrast microscope (*top panel*) and fixed and immuno-stained with an FITC-conjugated phalloidin. DAPI was used to stain nuclei (*bottom panel*). *B* and *C*, cells were fixed and co-immunostained with the anti-SDC-2 antibody and either the indicated anti-integrin antibodies (*B*) or the indicated anti-laminin chain antibodies (*C*). The results were visualized with either Texas Red-conjugated goat anti-rabbit antibodies or FITC-conjugated goat anti-mouse antibody. DAPI was used to stain nuclei. Data are presented as mean ± S.D. ∗∗, *p* < 0.01 *versus* HT29-Vec cells. N = 3. DAPI, 4′,6-diamidino-2-phenylindole; FITC, fluorescein isothiocyanate.

This SDC-2-mediated increase in laminin-332 appears to promote the formation of a stable coreceptor complex between SDC-2 and integrin α6β1, thereby improving the adhesion of HT29 cells to the ECM. Indeed, after 24 h, HT29-S2W cells showed greater attachment and spreading than controls, accompanied by extensive branched actin filament formation (Fig. 6*A*). Immunofluorescence staining further revealed that integrin α6 was specifically localized to cell-ECM contact sites on the basal surface, where it formed prominent adhesion complexes in HT29-S2W cells and SDC-2 overexpression also enhanced colocalization of SDC-2 with integrin α6 and β1 (Fig. 6*B*). Interestingly, however, colocalization between SDC-2 and laminin chains was relatively low (Fig. 6*C*), suggesting that most of the full-length laminin-332 upregulated by SDC-2 undergoes proteolytic cleavage of the α3 chain. The resulting processed laminin-332 matrix likely provides sufficient binding sites for independent yet cooperative interactions with integrin α6β1 and SDC-2.

### Both SDC-2 and integrin α6β1 regulate cell adhesion and migration of HT29 colon cancer cells

**Figure 7:**
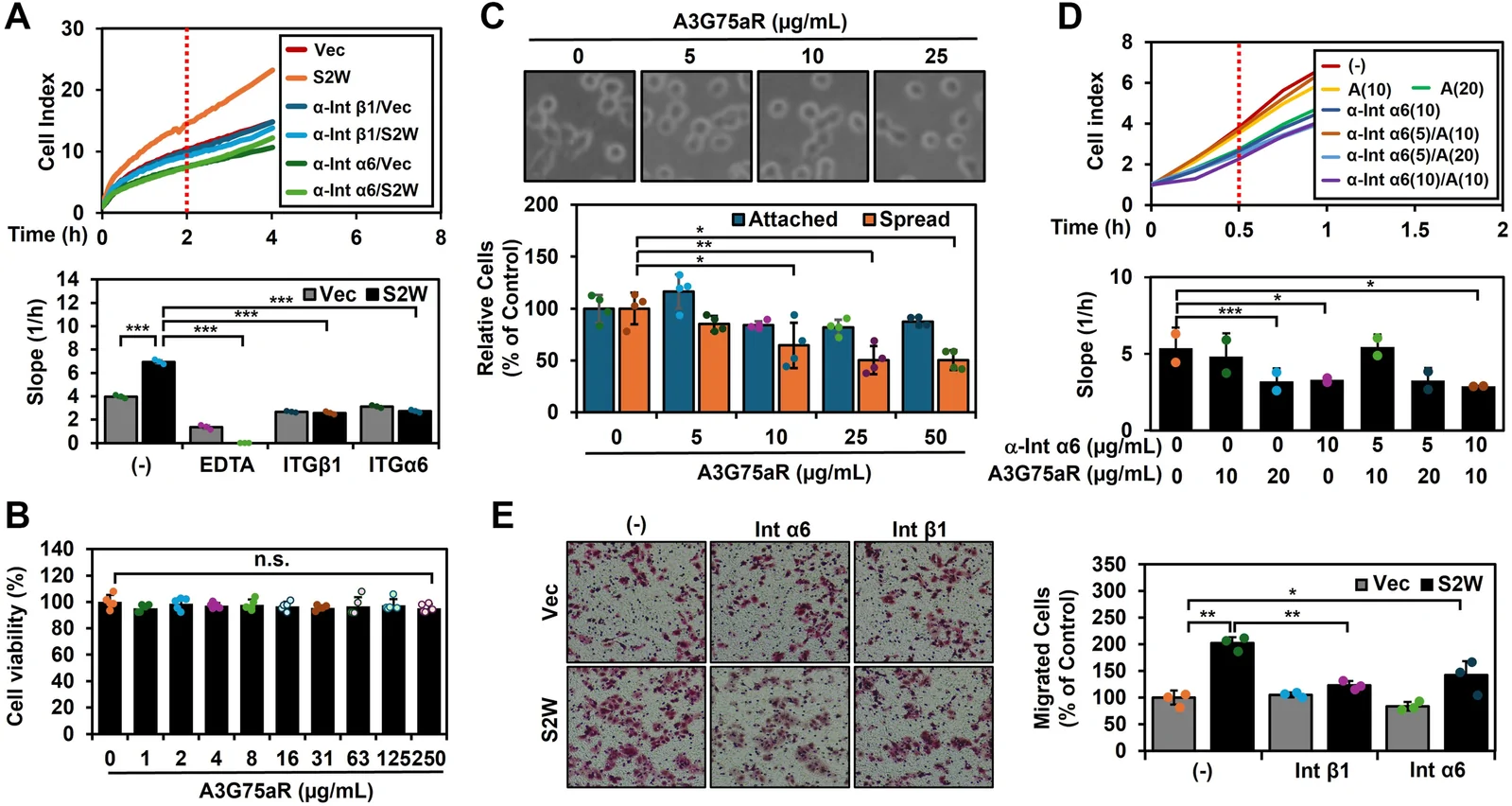
**Syndecan-2 and integrin α6β1 cooperatively regulate the adhesion and migration of HT-29 cells on full-length laminin-332.***A*, HT29-Vec and HT29-S2W cells (1.5 × 10^4^ cells/well) were seeded into E-plates precoated with full-length laminin-332 in the presence of either 5 mM EDTA or the indicated integrin antibody. Cell attachment was evaluated for 2 h with RTCA software. N = 3. *B*, HT29-S2W cells were treated with A3G75aR for 24 h, and cell viability was measured by MTT assay. *C*, HT29-S2W cells were seeded on full-length laminin-332-coated surfaces with increasing amount of A3G75aR. After 5 h, photographs were taken (*top panel*), and both attached and spread cells were counted. Results are expressed as means ± S.D. from three independent fields (*bottom panel*). N = 4. *D*, HT29-S2W cells (1.5 × 10^4^ cells/well) were seeded onto E-plates precoated with full-length laminin-332 in the presence of the indicated anti-integrin α6 antibody and/or A3G75aR. Cell adhesion was then evaluated using the xCELLigence system. N = 2. *E*, transwell migration assays were performed on full-length laminin-332-coated plates with the indicated anti-integrin antibody. The cells (5 × 10^5^ cells) were allowed to migrate for 18 h. The photographs were taken (*top panel*), and the migrated cells were quantified (*bottom panel*). Data are presented as mean ± S.D. from three randomly selected independent fields. N = 3. ∗, *p* < 0.05; ∗∗, *p* < 0.01; ∗∗∗, *p* < 0.001 *versus* the no-antibody (−) condition. MTT, 3-(4,5-dimethylthiazol-2-yl)-2,5-diphenyltetrazolium bromide.

To further investigate the mutual regulation between the two adhesion receptors, we examined whether blocking integrin α6β1 would affect SDC-2–mediated functions in HT29-S2W cells (Fig. 7). As shown in Figure 7*A*, SDC-2 expression increased cellular adhesion to full-length laminin-332; however, this enhancement was significantly inhibited by monoclonal antibodies targeting integrin α6 and β1 subunits. In addition, a synthetic peptide (A3G75aR), derived from the SDC-2 binding region within the laminin α3 LG4/5 module (33) did not affect cell viability up to 250 μg/ml (Fig. 7*B*), but significantly inhibited cell adhesion and spreading to full-length laminin-332 in a dose-dependent manner (Fig. 7*C*). Notably, combined treatment with the anti-integrin α6 antibody and A3G75aR further suppressed adhesion to full-length laminin-332 compared with either treatment alone, although no significant synergistic effect was observed (Fig. 7*D*). These results collectively underscore the critical role of both integrins α6β1 and specific laminin-332 domains in facilitating SDC-2-dependent cell adhesion. Similarly, transwell migration assays showed that enhanced migratory capacity induced by SDC-2 was significantly suppressed by anti-integrin α6 antibody, indicating that SDC-2’s migratory effect is largely dependent on integrin α6β1 (Fig. 7*E*). These data suggest that both SDC-2 and integrin α6β1 regulate cell adhesion and migration of HT29 colon cancer cells on laminin-332.

### SDC-2-laminin-332-integrin α6β1 axis promotes invasive motility of colon cancer cells

**Figure 8:**
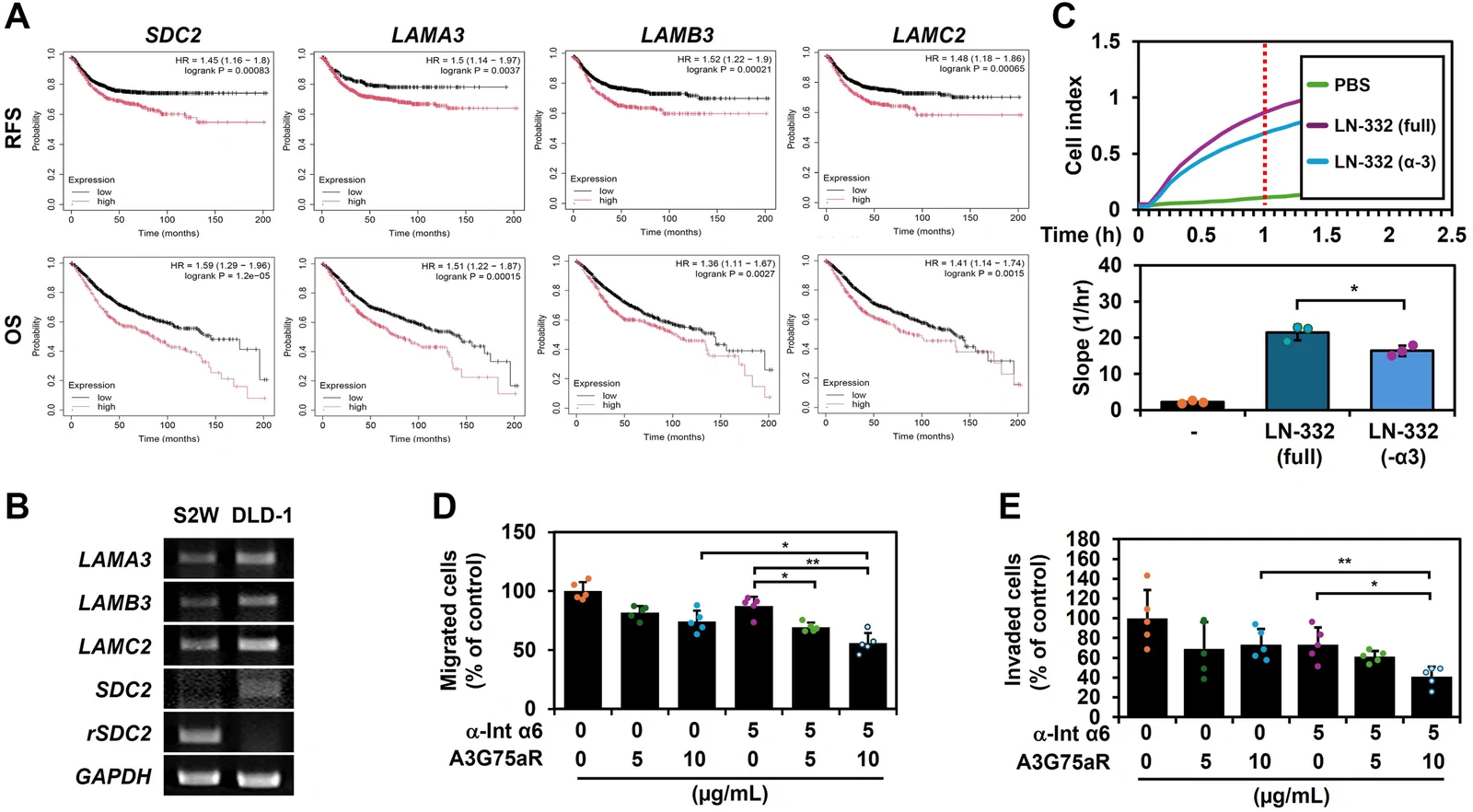
**Syndecan-2 and integrin α6β1 cooperatively regulate the invasive motility of DLD-1 cells.***A*, Kaplan–Meier survival analysis of relapse-free survival (RFS, *upper panels*) and overall survival (OS, *lower panels*) based on the expression levels of SDC-2 and laminin α3, β3, and γ2 chains. Patients were stratified into high- and low-expression groups, and survival differences were assessed using the log-rank test. Hazard ratios (HRs) with 95% confidence intervals (CIs) and corresponding *p*-values are indicated in each plot. *B*, total RNA was isolated from exponentially growing HT29-S2W or DLD-1 cells, and mRNA expression levels of SDC-2 and each laminin chain were analyzed by RT-PCR. GAPDH was used as a loading control. *C*, adhesion of DLD-1 cells to full-length laminin-332 or a truncated form lacking the α3 LG4/5 domain was evaluated using the xCELLigence system. N = 3. *D* and *E*, transwell migration and invasion assays were performed using DLD-1 cells treated with A3G75aR in the presence or absence of an anti-integrin α6 antibody. Data are presented as mean ± S.D. N = 5. ∗, *p* < 0.05; ∗∗, *p* < 0.01 *versus* control cells. SDC-2, syndecan-2.

Consistent with the enhanced migratory activity associated with SDC-2 and laminin-332 in colon cancer, analysis of the Kaplan–Meier plotter dataset for colon cancer patients revealed that high expression of both SDC-2 and the laminin α3, β3, and γ2 chains was significantly correlated with poor relapse-free survival and overall survival (Fig. 8*A*). Consistently, metastatic DLD-1 cells exhibited elevated expression of both SDC-2 and laminin-332 (Fig. 8*B*) and attached more efficiently to full-length laminin-332 than to the truncated form (Fig. 8*C*). Furthermore, treatment with A3G75aR significantly reduced both migration and invasion of DLD-1 cells in a dose-dependent manner and further enhanced the inhibitory effects of an anti-integrin α6 antibody on cell migration and invasion in a dose-dependent manner (Fig. 8, *D* and *E*). Taken together, these findings suggest that both SDC-2 and integrin α6β1 contribute to the regulation of colon cancer cell adhesion and migration, and that the SDC-2–laminin-332–integrin α6β1 axis plays a key role in promoting colon cancer cell adhesion and motility.

## Discussion

Although SDC-2 is known to interact with laminin-332 (22), the functional significance of this interaction in colon cancer progression remains unclear. Notably, elevated expression of the laminin γ2 and β3 chains at the invasive front of carcinoma cells has been identified as a poor prognostic marker in colon cancer (12). However, the specific contribution of SDC-2 to laminin-332–mediated processes has not been thoroughly investigated. In this study, we identified a previously uncharacterized regulatory axis involving SDC-2, laminin-332, and integrin α6β1 that cooperatively promotes colon cancer cell motility.

Our results demonstrate that laminin-332 is a key adhesive substrate for HT29 cells *via* integrins α6β1 and α3β1 (Figs. 1 and 2). SDC-2 overexpression enhances this adhesion, likely by recruiting integrin α6β1 to the cell–ECM interface (Figs. 3 and 6). Mechanistically, SDC-2 overexpression enhances its interaction with integrin α6β1, as shown by increased proximity (Fig. 4*D*), and promotes integrin colocalization at the basal membrane (Fig. 6). This interaction likely facilitates integrin activation and cell motility in a laminin-332–dependent manner. SDC-2 also increases expression and ECM deposition of laminin-332 α3, β3, and γ2 chains (Fig. 5), enriching the matrix and enabling greater integrin α6β1 engagement. Notably, despite promoting integrin–ECM association, SDC-2 does not directly colocalize with laminin-332 (Fig. 6). These results suggest that most of the full-length laminin-332 upregulated by SDC-2 undergoes proteolytic cleavage of its α3 chain’s C-terminal LG4/5 domain to generate the processed form of laminin-332. Thus, SDC-2 increases the availability of laminin-332 and promotes integrin activation without directly bridging laminin-332 and integrin α6β1. Instead, the increased deposition of laminin-332 in the ECM likely facilitates greater adhesive and migratory potential through both SDC-2 and integrin α6β1 in colon cancer cells. While both SDC-2 and laminin-332 have previously been implicated in cancer progression and metastasis, their functional interplay has remained poorly defined. Our findings address this gap by showing that SDC-2 acts as a scaffold and modulator of integrin-mediated signaling, organizing an ECM environment conducive to cell migration.

Functionally, SDC-2 overexpression led to increased adhesion-dependent migration toward laminin-332. This migration was significantly suppressed by either α6 integrin–blocking monoclonal antibodies or a synthetic peptide (A3G75aR) which targets the laminin α3 LG45 domain, the SDC-2 binding site. Notably, the migration and invasion of highly metastatic DLD-1 cells, which express high levels of both SDC-2 and laminin-332 were cooperatively inhibited by the combination of antibody and the peptide (Fig. 8). These results support the notion that both SDC-2 and integrin α6β1 mediated function cooperatively to mediate cell adhesion and motility on laminin-332, with SDC-2 primarily enhancing integrin α6β1 dependent adhesion. Although our data show that the antibody against integrin β1 inhibited HT29 adhesion to laminin-332 by nearly 70%, suggesting that integrin α6β1 is primarily involved in this regulation, we cannot rule out the possibility that other integrins, such as α6β4—which mainly mediates stable interactions with laminin-332 through hemidesmosomes—may also contribute to this process.

Despite the central role of integrins in cancer cell adhesion and migration, therapeutic strategies targeting integrins alone have achieved limited success, due in part to functional redundancy among integrin subtypes, variability in expression, and context-specific roles during tumor progression (34). Consequently, several efforts have shifted toward targeting integrin regulatory mechanisms mediated by SDCs. For example, Synstatin inhibits tumorigenesis by disrupting SDC-1 interactions with integrins αvβ3 and αvβ5 (35), while SDC-4 inhibition has shown potential in blocking FGF and integrin signaling during capsular opacification (36). Similarly, the recombinant CBD-HepⅡ peptide (CH50) was shown to suppress lung metastasis by downregulating both integrin αvβ3 and SDC-1 (37). Given the growing evidence implicating SDC-2 in colon cancer progression (38), along with our findings that high expression of both SDC-2 and laminin-332 is associated with poor relapse-free and overall survival (Fig. 8*A*), and that treatment with A3G75aR enhances the inhibitory effects of an anti-integrin α6 antibody on cell migration and invasion in a dose-dependent manner (Fig. 8, *C* and *D*), we propose that targeting the SDC-2–integrin α6β1 axis on laminin-332 may represent a promising therapeutic strategy.

**Figure 9:**
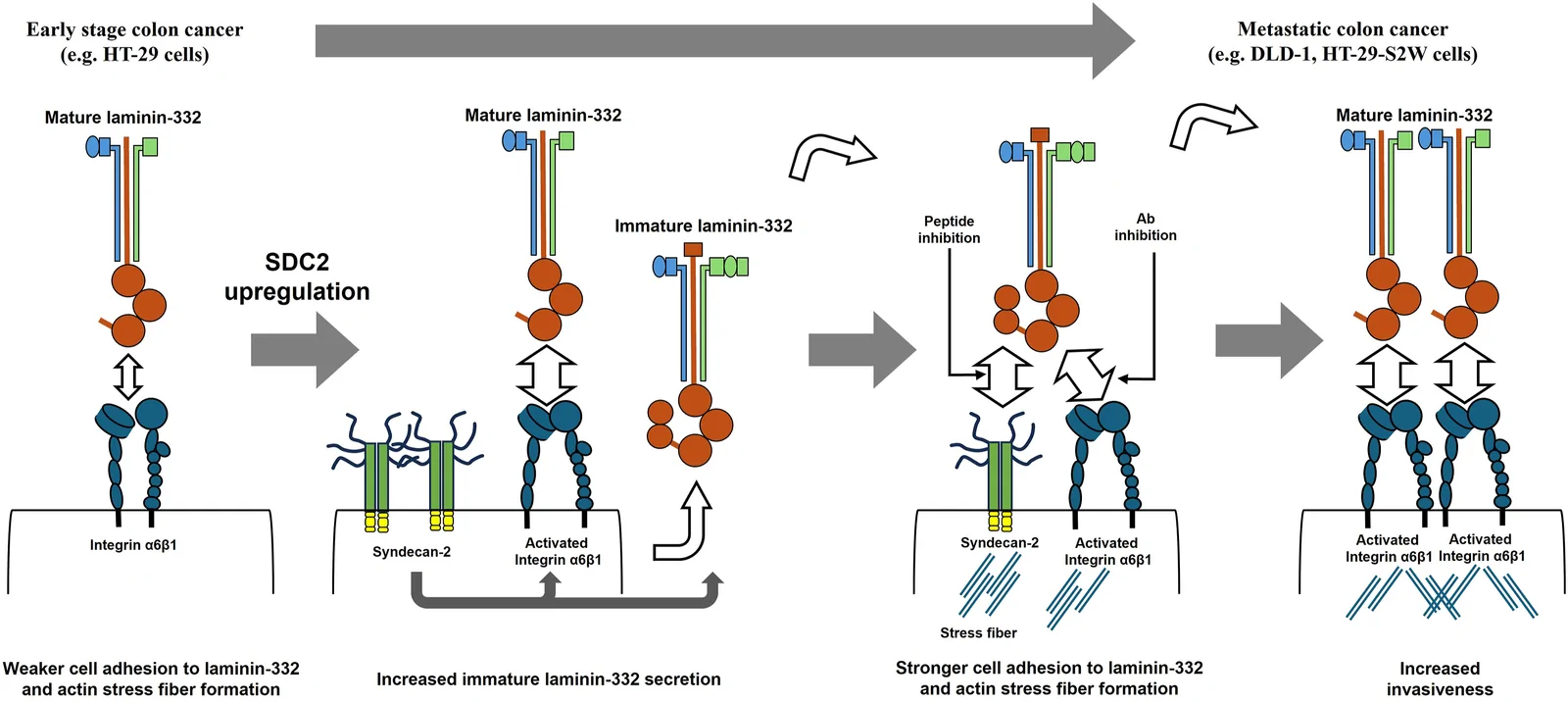
**Hypothetical mechanism of the syndecan-2–integrin α6β1–laminin-332 regulatory axis in controlling the invasive motility of colon cancer cells.** Early-stage colon cancer cells (*e.g.*, HT29 cells) express low levels of SDC-2 and therefore exhibit limited migratory and invasive capacity. During cancer progression; however, increased SDC-2 expression promotes the activation of integrin α6β1, which enhances both the expression and deposition of immature laminin-332 containing the SDC-2–binding LG4/5 modules in the ECM. This leads to the formation of a stronger adhesion complex composed of SDC-2 and integrin α6β1 on laminin-332. Within this complex, SDC-2 and integrin α6β1 bind independently to laminin-332, but also mutually modulate each other’s interactions with immature laminin-332, thereby strengthening laminin-332–mediated cell adhesion. In addition to its role as an adhesion receptor promoting cell migration, SDC-2–induced increases in immature laminin-332 also facilitate its subsequent processing into mature laminin-332, which further enhances the invasive motility of colon cancer cells such as DLD-1. SDC-2, syndecan-2.

In summary, we have identified an SDC-2–integrin α6β1–laminin-332 regulatory axis that governs colon cancer cell adhesion, migration, and potentially metastatic behavior. SDC-2 enhances integrin α6β1-mediated adhesion by promoting laminin-332 deposition and recruiting integrin α6β1 to adhesion sites. In this context, SDC-2 primarily functions as a structural scaffold and regulatory modulator, rather than as a direct ligand for laminin-332 (Fig. 9). Dual targeting of SDC-2 and integrin α6β1 effectively inhibited the migration of SDC-2–overexpressing HT29 cells and the invasive behavior of DLD-1 cells. In contrast to prior studies that examined SDC-2 or integrin roles in isolation, our work is the first to demonstrate a cooperative mechanism linking SDC-2, laminin-332, and integrin α6β1 in colon cancer cells. While further studies are needed to fully elucidate the molecular mechanisms of SDC-2’s promigratory effects, our findings highlight this axis as a potential target for therapeutic intervention in metastatic colorectal cancer.

## Experimental procedures

### Materials

A monoclonal antibody capable of recognizing human, rat, and mouse SDC-2 was produced by AdipoGen Inc. (Incheon) using the Fc-fused extracellular domain of SDC-2 (7). Monoclonal antibodies against human integrins α2 (P1E6), α3 (P1B5), α4 (P4G9), α5 (P1D6), α6 (P5G10), αV (P3G8), and β1 (AIIB2) were purified using Protein G Sepharose (Cytiva) from conditioned media of hybridoma cells purchased from the Developmental Studies Hybridoma Bank (22). The following monoclonal antibodies were used: anti-laminin α3A chain (Funakoshi Co., Ltd), anti-laminin β3 (sc-133178), anti-laminin γ2 (sc-25341) chain, anti-fibronectin and anti-β-actin (Santa Cruz Biotechnology), and anti-integrin β1 (TS2/16) (BioLegend). Rabbit polyclonal antibodies against laminin α3A chain (1110+), laminin β3 (1111+), and laminin γ2 (1097) were generated using antigens corresponding to their respective domains (LEc, LM/LE, and LE4-6) and were kindly provided by Dr Takako Sasaki (Oita University). Anti-integrin β3 (#4702), β4 (#14803) and β5 (#4708) antibodies (polyclonal) were obtained from Cell Signaling Technology. In addition, rabbit polyclonal antibodies against integrin α3, α6, and β1 were purchased from Bicell Scientific. The laminin-332 lacking the α3 LG4/5 domain was purchased from OYC America and the full-length, unprocessed laminin-332 containing the LG4/5 domain was purchased from BioLamina. Fibronectin was obtained from Invitrogen. iMatrix-511 (LM-511E8) was purchased from AMSBIO (Oxfordshire). Mouse EHS laminin-111 was obtained from BD Biosciences, and Matrigel was purchased from Collaborative Biomedical Products. Gelatin and type I collagen were purchased from Sigma-Aldrich. EDTA solution (pH 8.0) was obtained from LPS Solution. The A3G75aR peptide, corresponding to a laminin α3 chain LG4/5 domain sequence (33), was chemically synthesized and used as an inhibitor of the SDC-2 binding site on laminin-332. The antibodies used in the assay are listed below (Table 1).

**Table 1:** Antibodies used for immunodetection and functional assays

Antibody		Application				
Name	Clone or Cat. No.	If	Flow cytometry	PLA	Western blotting	Adhesion blocking
Integrin α2	P1E6	Fig. 2*B*	Fig. 2*C*			
Integrin α3	P1B5	Fig. 2*B*	Fig. 2*C* and 4*B*			Fig. 2*E*
	BiCell Scientific			Fig. 4*D*		
Integrin α4	P4G9	Fig. 2*B*	Fig. 2*C*			
Integrin α5	P1D6	Fig. 2*B*	Fig. 2*C*			
Integrin α6	P5G10	Fig. 2*B*	Fig. 2*C*			Figs. 2, *E*, 7, *A*, *D*, *E* and 8, *D*, *E*
	BiCell Scientific	Figs. 4*C* and 6*B*	Fig. 4*B*	Fig. 4*D*		
Integrin αv	P3G8	Fig. 2*B*	Fig. 2*C*			
Integrin β1	TS2/16	Fig. 2*B*	Fig. 2*C* and 4*B*			
	AIIB2					Figs. 2, *E* and 7, *A*, *D*, *E*
	BiCell Scientific	Figs. 4*C* and 6*B*		Fig. 4*D*		
Integrin β3	#4702	Fig. 2*B*				
Integrin β4	#14803	Fig. 4*C*	Fig. 4*B*	Fig. 4*D*		
Integrin β5	#4708	Fig. 2*B*				
Laminin α3A	FDA-0024	Fig. 5, *C* and *E*			Fig. 5*D*	
	LEc1110+	Fig. 6*C*				
Laminin β3	sc-133178	Fig. 5, *C* and *E*			Fig. 5*D*	
	LM/LE 111+	Fig. 6*C*				
Laminin γ2	sc-25341	Fig. 5, *C* and *E*			Fig. 5*D*	
	LE4-6 1097+	Fig. 6*C*				
Fibronectin	sc-84223	Fig. 5*E*				

### Cell culture and generation of stable cell lines

The human colon adenocarcinoma cell lines HT29 and DLD-1 were purchased from the American Type Culture Collection ATCC. Upon receipt, low-passage cell stocks were cryopreserved. For all experiments, cells were thawed and used within 20 passages to maintain phenotypic stability and authenticity. HT29 cells were maintained in McCoy’s 5A complete medium (Welgene), while DLD-1 cells were cultured in DMEM complete medium (Hyclone), both supplemented with 10% (v/v) fetal bovine serum (FBS; Hyclone) and 0.1% gentamicin (50 mg/ml; Sigma-Aldrich). Cells were incubated at 37 °C in a humidified atmosphere containing 5% CO_2_. To generate stable SDC-2–expressing cell lines, HT29 cells (1 × 10^5^) were transfected with 1 μg of either empty pcDNA3.1 vector (control, V) or pcDNA3.1-FLAG-SDC2 (WT, 2W) (7) using the Viva Magic transfection reagent (Vivagen), following the manufacturer’s protocol. Transfected cells were selected in McCoy’s 5A medium containing 400 μg/ml G418 (EMD Biosciences) for 4 weeks. Surviving clones were individually isolated and screened by fluorescence-activated cell sorting and RT-PCR.

### RT-PCR

Total RNA was isolated from cells using an easy-BLUE kit (Intron). The RNA was extracted with chloroform and precipitated with isopropanol. The RNA pellet was washed with 75% ethanol and resuspended in DEPC-treated water. Approximately 3 μg of RNA was used to generate cDNA using AMV Reverse Transcriptase (Cat# M5108) and Random Primer (Cat# C1181) (both from Promega US). Aliquots of the resulting cDNAs were amplified using specific primers (Table 2). After an initial denaturation at 94 °C for 5 min, the samples were subjected to 30 cycles of denaturation at 94 °C for 30 s, annealing at 55 °C for 30 s, and extension at 72 °C for 60 s. Human GAPDH was amplified as an internal control. The generated PCR products were separated by 1% agarose gel electrophoresis. The gel was imaged using a Davinchi Gel imaging System (Davinchi-K).

**Table 2:** Primer lists

Gene	Forward primer	Reverse primer
*rSDC2*	ATGCGGGTACGAGCCACGTC	CGGGAGCAGCACTAGTGAGG
*hSDC2*	ACATCTCCCCTTTGCTAACGGC	TAACTCCATCTCCTTCCCCAGG
*hLAMA1*	GTCAGCGACTCAGAGTGTTTG	AACTTGGGTGAAAGATCGTCAG
*hLAMA2*	GAACCCGCAGTGTCGAATCT	GGGGAGTTAGCTGCCTTCA
*hLAMA3*	CGTGAGGCTGAACTCCAAGT	CTGGATGTGGCTCCTTTGGT
*hLAMA4*	GATGCCGTACTCTGCTGGTT	AGGCTGAGCTCAAAGCCATT
*hLAMA5*	GGTGTGTCTCTGCGTGACAA	CCCCGACGTAGAAA
*hLAMB1*	AGGAACCCGAGTTCAGCTAC	CACGTCGAGGTCACCGAAA
*hLAMB2*	GCCCTGGGAACTTCGACTG	GGAAGCACTTCTTTTCGTCCTG
*hLAMB3*	TCCTCTTGTGTTTTGCCCTG	CTGCCTGGAGTCACACTTG
*hLAMC1*	GAGGCAAGATATCGCCGTGA	GTATCTCGCCTGTCCACTCG
*hLAMC2*	TGGAGAACGCTGTGATAGGTGTCG	GCAGTGAATCCCATCAGTGTT
*hLAMC3*	CTCTGCCTCAGAAGTCCCG	TGTGTAAGTCTTGGTGAGCCCAC
*hITGA2*	GGGCATTGAAAACACTCGAT	TCGGATCCCAAGATTTTCTG
*hITGA3*	TGGGAGCTGTTTATTGGTCG	GGGCCTAGAGGTGGAGTTCT
*hITGA4*	TGGCTCCCAATGTTAGTGTGGA	TGCGCAACATTCTCATCCTTG
*hITGA5*	CCTCCCAATTTCAGACTCCC	ACAAGGGTCCTTCACAGTGC
*hITGA6*	GACTTGAAAGAAATGGTGAATGC	TAGCACCTGTTTGCTGTTGC
*hITGA7*	GGGCCAACATCACAGTGAAG	CCCCAGTTGTTCCCTCAGGAT
*hITGAV*	CAGGCTTGCAACCCATTCTT	TGGTGCACACTGAAACGAAG
*hITGB1*	AATGTTTCAGTGCAGAGC	TTGGGATGATGTCGGGAC
*hITGB3*	ACCACTGATGCCAAGACTCA	ACGCACTTCCAGCTCTACTT
*hITGB4*	TCACCAACCTGTACCCGTAT	AAGCAGCATCCGGTTCTTAG
*hITGB5*	GTGGAGTTGTCAGTCTGGGA	TTGTGGTACACGCTCTGGTT
*hGAPDH*	CCTCAAGATCATCAGCAAT	CCATCCACAGTCTTCTGGGT

### Immunoblotting

HT29 cells (5 × 10^5^ cells/well) were seeded on six well plate and incubated for 48 h at 37 °C in a 5% CO_2_ atmosphere. Cells were then washed with PBS and lysed with RIPA buffer (50 mM Tris (pH 8.0), 150 mM NaCl, 1% Nonidet P-40, 10 μM NaF, and 2 μM Na_3_VO_4_) containing a protease inhibitor mixture (1 μg/ml antipain, 1 μg/ml pepstatin A, 5 μg/ml leupeptin, and 20 μg/ml phenylmethylsulfonyl fluoride). Cell lysates were clarified by centrifugation at 13,000 rpm for 15 min at 4 °C, denatured with SDS-PAGE sample buffer, boiled at 95 °C for 5 min, and analyzed by SDS-PAGE. Proteins were transferred to 0.45-μm nitrocellulose membranes (GE Healthcare) and probed with specific antibodies. Signals were detected using an Odyssey CLx imager (LI-COR Biosciences), and the data were analyzed with the Image Studio Lite Software (Li-Cor Biosciences).

### Flow cytometry

HT29 cells (5 × 10^5^ cells/well) were washed with PBS, then released using 5% FBS and 1 mM EDTA in PBS, collected by centrifugation, resuspended in PBS, and incubated with specific antibodies with 5% FBS in PBS for overnight at 4 °C. The cells were washed three times with PBS containing 0.05% Tween-20 and incubated with FITC-conjugated anti-mouse IgG (Thermo Fisher Scientific) in PBS containing 10% FBS for 1 h at RT. The expression of Integrins and SDC-2 at cell surface was evaluated by flow cytometry counts of 1 × 10^4^ cells (BD LSRFortessa).

### Monitoring of cell attachment

Cell adhesion was monitored in real time using an xCELLigence system (Roche Diagnostics GmbH). The bottoms of E16 plates (ACEA Biosciences) were coated with 10 μg/ml of ECM at 37 °C for 1 h. After washing with PBS, the plates were blocked with 0.2% heat-inactivated bovine serum albumin (BSA) in PBS at 25 °C for 1 h. Plates were then washed again with PBS, filled with 50 μl of culture medium per well, and incubated at 37 °C with 5% CO_2_ for 10 min. After removal of the medium, HT29-Vec and HT29-S2W cells (1.5 × 10^4^ cells/well) were added to each well, either alone or in combination with the indicated anti-integrin antibody or A3G75aR peptide. Following a 10-min preincubation, the plates were transferred to the RTCA DP Analyzer, and real-time cell adhesion was monitored at 3-min intervals under 5% CO_2_. Data were subsequently normalized and analyzed using the RTCA software.

### Cell spreading and adhesion assay

For the adhesion assay, 96-well microtiter plates (Thermo Fisher Scientific) were coated with recombinant laminin-332 and subsequently blocked with PBS containing 0.5% heat-inactivated BSA. Dissociated cells were resuspended in medium and plated at a density of 4 × 10^4^ cells/well. After incubation at 37 °C for 30 min, adherent cells were stained using the Diff-Quik staining kit (Sysmex). Stained cells were quantified using an image cytometer (BZ-X800, Keyence). To identify the receptors involved in laminin-332 binding, cells were preincubated with the indicated integrin antibodies or the A3G75aR peptide for 10 min at RT before plating.

For the spreading assay, 96-well plates (Thermo Fisher Scientific) were coated with recombinant laminin-332 (10 μg/ml, 50 μl/well) and incubated at 25 °C for at least 1 h. After washing with PBS, the wells were blocked with 0.2% heat-inactivated BSA for 1 h and washed again with PBS. Cells were then detached, resuspended in medium, and plated at a density of 4 × 10^4^ cells/well onto the ECM-coated wells. After incubation at 37 °C for various time points, cell spreading was assessed by phase-contrast microscopy using a digital camera-equipped microscope (Zeiss).

### Immunofluorescence analysis

HT29 cells (2 × 10^5^ cells/well) were seeded onto glass coverslips placed in 12-well plates and incubated for 72 h at 37 °C in a humidified atmosphere containing 5% CO_2_. To assess the effects of laminin-332, HT29 cells (1 × 10^5^ cells/well) were seeded onto glass coverslips precoated with recombinant laminin-332 in 24-well plates and incubated for either 5 or 24 h under the same conditions. Following incubation, cells were rinsed three times with PBS and fixed with 3.5% paraformaldehyde in PBS at RT for 10 min. After fixation, cells were washed three times with PBS, blocked with 0.5% BSA in PBS for 1 h at RT, and then incubated with primary antibodies overnight at 4 °C. The next day, cells were washed with PBS and incubated for 1 h at RT with FITC-conjugated mouse secondary antibodies and Texas Red-conjugated rabbit secondary antibodies (Thermo Fisher Scientific). After a final PBS wash, the coverslips were mounted on glass slides using a mounting medium containing 4′,6-diamidino-2-phenylindole. Fluorescence images were acquired using a confocal fluorescence microscope (Carl Zeiss).

### Transwell migration assay

For the migration assay, 24-well Transwell plates with 8-μm pore size polycarbonate membranes (Costar) were used. Each membrane was coated with laminin-332 (10 μg/ml) and air-dried at 25 °C for 1 h. The Transwell inserts were then placed into 24-well plates containing chemoattractant medium—20% FBS for HT29 cells or 10% FBS for DLD-1 cells. HT29 cells (5 × 10^5^) suspended in medium containing 5% FBS, or DLD-1 cells (1 × 10^5^) in serum-free medium, were added to the upper chambers. The plates were incubated at 37 °C in a 5% CO_2_ incubator for 18 h (HT29) or 16 h (DLD-1). After incubation, non-migrated cells on the upper side of the membrane were removed, and the migrated cells on the lower side were fixed, stained with hematoxylin and 0.5% eosin, and counted under a microscope. For the invasion assay, Transwell membranes were precoated with gelatin (10 μg/ml) on the lower side and Matrigel (diluted 1:30 in serum-free medium; BD Biosciences) on the upper side to mimic the ECM barrier.

### Cell proliferation assay

Cell proliferation was assessed using the 3-(4,5-dimethylthiazol-2-yl)-2,5-diphenyltetrazolium bromide assay. HT29 cells were harvested using 0.05% trypsin-EDTA and seeded into 96-well plates at a density of 2 × 10^4^ cells/well. The cells were incubated for 24 h at 37 °C in a humidified atmosphere containing 5% CO_2_. Following incubation, A3G75aR was added at varying concentrations up to 250 μg/ml to each well. After an additional 24-h incubation, 100 μl of medium containing 0.5 mg/ml 3-(4,5-dimethylthiazol-2-yl)-2,5-diphenyltetrazolium bromide (Sigma-Aldrich) was added to each well, and the plates were incubated for 1 h at 37 °C. The medium was then removed, and 100 μl of acidic isopropanol (90% isopropanol, 0.5% SDS, 25 mM NaCl) was added to solubilize the formazan crystals. Absorbance was measured at 570 nm using a 96-well microplate reader (Dynatech), and mean values were calculated for each treatment group.

### Isolation of ECM

Cells (1 × 10^6^ HT29 cells) were seeded and incubated for 72 h at 37 °C in a 5% CO_2_ atmosphere. The culture medium was removed, and the cells were washed three times with PBS. Ammonium hydroxide (20 mM) was added and incubated for 5 min at RT. The insoluble ECM layer was washed four more times to ensure complete removal. For examination of the ECM by immunofluorescence, the ECM was fixed with 3.5% paraformaldehyde in PBS. For examination of the ECM by immunoblotting, a preheated SDS-PAGE sample buffer containing 100 mM dithiothreitol was added to the plate. The ECM was thoroughly scraped with a cell scraper and analyzed by SDS-PAGE.

### Proximity-ligation assay

HT29 cells on coverslips were fixed with 3.5% paraformaldehyde in PBS at RT and briefly washed three times with PBS. The cells were permeabilized with 0.1% Triton X-100 in PBS for 15 min and washed three times with PBS. After washing, the PLA was performed according to the manufacturer's instructions (NaveniFlex MR, Navinci Diagnostics). Briefly, cells were blocked with blocking solution at 37 °C for 1 h, then incubated with primary antibodies overnight at 4 °C. After washing with 1 × TBST, cells were incubated with Navenibody M1 and R1 for 1 h at 37 °C, followed by two washes with TBST. Enzyme 1 was added for 30 min at 37 °C, and after washing, Enzyme two was applied for 90 min at 37 °C. Cells were then washed with 1 × TBS. Coverslips were mounted using 4′,6-diamidino-2-phenylindole-containing mounting medium, and images were acquired using a confocal fluorescence microscope (Carl Zeiss).

### Statistical analysis

All data are presented as the mean ± SD. Differences between groups were tested for statistical significance using Student’s *t* test and considered significant at ∗, *p* < 0.05, ∗∗, *p* < 0.01, or ∗∗∗, *p* < 0.005.

## Data availability

All raw data will be available upon request from the corresponding authors.

## Conflict of interest

The authors declare that they have no conflicts of interest with the contents of this article.
